# Genomic Landscapes of Noncoding RNAs Regulating *VEGFA* and *VEGFC* Expression in Endothelial Cells

**DOI:** 10.1128/MCB.00594-20

**Published:** 2021-06-23

**Authors:** Isidore Mushimiyimana, Vanesa Tomas Bosch, Henri Niskanen, Nicholas L. Downes, Pierre R. Moreau, Kiley Hartigan, Seppo Ylä-Herttuala, Nihay Laham-Karam, Minna U. Kaikkonen

**Affiliations:** a A. I. Virtanen Institute for Molecular Sciences, University of Eastern Finland, Kuopio, Finland; b University of Colorado Boulder, Boulder, Colorado, USA; c Heart Center and Gene Therapy Unit, Kuopio University Hospital, Kuopio, Finland

**Keywords:** VEGF, VEGFC, atherosclerosis, eRNA, lncRNA

## Abstract

Vascular endothelial growth factors (VEGFs) are best known as key regulators of angiogenesis and lymphangiogenesis. Although VEGFs have been promising therapeutic targets for various cardiovascular diseases, their regulatory landscape in endothelial cells remains elusive. Several studies have highlighted the involvement of noncoding RNAs (ncRNAs) in the modulation of *VEGF* expression. In this study, we investigated the role of two classes of ncRNAs, long ncRNAs (lncRNAs) and enhancer RNAs (eRNAs), in the transcriptional regulation of *VEGFA* and *VEGFC*. By integrating genome-wide global run-on sequencing (GRO-Seq) and chromosome conformation capture (Hi-C) data, we identified putative lncRNAs and eRNAs associated with *VEGFA* and *VEGFC* genes in endothelial cells. A subset of the identified putative enhancers demonstrated regulatory activity in a reporter assay. Importantly, we demonstrate that deletion of enhancers and lncRNAs by CRISPR/Cas9 promoted significant changes in *VEGFA* and *VEGFC* expression. Transcriptome sequencing (RNA-Seq) data from lncRNA deletions showed downstream factors implicated in *VEGFA-* and *VEGFC*-linked pathways, such as angiogenesis and lymphangiogenesis, suggesting functional roles for these lncRNAs. Our study uncovers novel lncRNAs and eRNAs regulating *VEGFA* and *VEGFC* that can be targeted to modulate the expression of these important molecules in endothelial cells.

## INTRODUCTION

The vascular endothelial growth factor (VEGF) family is composed of 5 members in mammals: VEGF-A, -B, -C, and -D and placental growth factor (PlGF) (1–5), which exert their functions through three tyrosine kinase receptors: VEGFR-1 (Flt-1), VEGFR-2 (KDR/FLK-1), and VEGFR-3 (Flt4) (6–8). VEGFs play a role in blood and lymph vessel development and homeostasis, and their function is critical in the neural and hematopoietic systems, bone development, and mammalian reproductive organs (9). Particularly, *VEGFA* is involved in angiogenesis and vasculogenesis (10), and *VEGFC* mediates lymphangiogenesis, although it has been shown that it also has angiogenic activity (11, 12).

Expression of *VEGF*s is induced in hypoxia in order to improve oxygen delivery through transcriptional regulation by hypoxia-inducible transcription factor 1α (HIF1α) (13). Additionally, other external signals, such as growth factors and interleukins (ILs), can induce *VEGF* expression (14). Due to their crucial role in regulating endothelial cell behavior, VEGFs are therapeutic targets for many diseases, such as cardiovascular diseases (15) and cancer (16).

Although the field of angiogenic therapy for the treatment of cardiovascular diseases is being continuously expanded, clinical trials with angiogenic proteins have not yet been successful (15). Hence, new therapeutic avenues for angiogenesis are required. Moreover, little is known about the regulatory landscape of endothelial cells in the vasculature. Thus, a better understanding of the angiogenic regulatory network in endothelial cell function is needed for therapeutic advances. The angiogenic regulatory network can encompass many layers of regulation, and one of these is at the transcriptional level. In recent years, we have come to appreciate the complexity of transcriptional regulation and the role of noncoding RNAs (ncRNAs) in this process (17).

Protein-coding genes comprise less than 3% of the human genome, while the remaining 97% was once thought to be transcriptionally inactive. However, advances in sequencing techniques have revealed that the majority of the human genome is transcribed as noncoding RNA, and importantly, these regions also harbor the majority of disease-linked variants (18). The biggest class of ncRNA is comprised of long ncRNAs (lncRNAs), which are commonly defined as noncoding transcripts longer than 200 nucleotides (nt) (19). lncRNAs are a diverse class of transcripts that may overlap genes and be intergenic (lincRNAs) (20) and that can also originate from enhancer regions (eRNAs) (21). Although the estimated number of lncRNAs in humans is about 100,000, the functions of the vast majority remain uncharacterized (22). However, the use of next-generation sequencing (NGS) techniques allows identification of novel ncRNAs.

Recent studies have suggested that lncRNAs act to regulate genes in *cis* and in *trans* in a transcriptional or posttranscriptional manner during development, differentiation, and human diseases (17, 23). lncRNAs can regulate transcriptional activity in *cis* by recruiting chromatin-regulating complexes and thereby regulating the chromatin state (24). Furthermore, the *cis*-regulatory effect can be due to the transcription event itself or enhancer-like activities of the DNA sequence (25). *trans*-acting lncRNAs can recruit chromatin modifiers affecting the expression of multiple genes (26) and regulate transcription factor binding and RNA polymerase activity (27). Moreover, *trans*-acting lncRNAs can also act at a posttranscriptional level, regulating alternative splicing (28), mRNA decay (29), and regulation of translation (30). Additionally, studies have shown that lncRNAs participate in genomic organization (31).

One class of lncRNAs that are of particular interest is the enhancer RNAs. These RNAs arise from enhancers, which are defined as DNA sequences that increase the expression of protein-coding genes and can function in a cell type-specific manner (32), independently of their orientation and position to the target gene (33). Enhancer RNAs are usually less than 2 kb in length, are bidirectionally transcribed, and lack either splicing or polyadenylation (34). Some studies have shown that eRNAs regulate gene expression (35), promote enhancer-promoter looping (36), and bind to chromatin modifiers (37), hence remodeling chromatin conformation. Furthermore, eRNAs can recruit transcription factors (38) and regulate transcriptional machinery (39). Genetic variance in enhancers can thus modify the binding of transcription factors, leading to improper gene expression and susceptibility to disease (40).

The aim of this study was to decipher novel noncoding RNAs participating in the regulation of *VEGF*s in endothelial cells in order to provide a better understanding of this complex angiogenic regulatory network. Regulating *VEGF*s would be important to reverse neovascularization of atherosclerotic plaques, while revascularization of ischemic tissues would represent an important therapeutic strategy for treating atherosclerotic complications. Therefore, novel insights into the transcriptional landscape of *VEGF* genes may prove valuable for future therapeutic applications. By using next-generation sequencing data from endothelial cells, we identified novel eRNAs and lncRNAs that interact with *VEGFA* and *VEGFC* promoters. We discovered six and three putative enhancers for *VEGFA* and *VEGFC,* respectively, and identified active enhancers using reporter assays. We also demonstrated that deletion of *VEGFA* enhancer 5 (*VEGFA-E5*) and *VEGFC* enhancer 3 (*VEGFC-E3*) decrease *VEGFA* and *VEGFC* gene expression, respectively. Furthermore, global run-on sequencing (GRO-Seq) data allowed us to identify one lncRNA transcribed antisense to the *VEGFA* gene (*VEGFA-LNC*) and one lncRNA located 120 kb upstream of *VEGFC* (*VEGFC-LNC*) in human umbilical vein endothelial cells (HUVECs). Deletion of *VEGFA-LNC* increased *VEGFA* expression, while deletion of *VEGFC-LNC* decreased *VEGFC* expression. Finally, we studied the genome-wide effects of these deletions using transcriptome sequencing (RNA-Seq) and found that they affected gene ontology (GO) pathways implicated in endothelial cell biology. Thus, our results uncover novel noncoding RNAs participating in the regulation of *VEGF* gene expression.

## RESULTS

### Identification and characterization of *VEGFA* enhancers.

**FIG 1:**
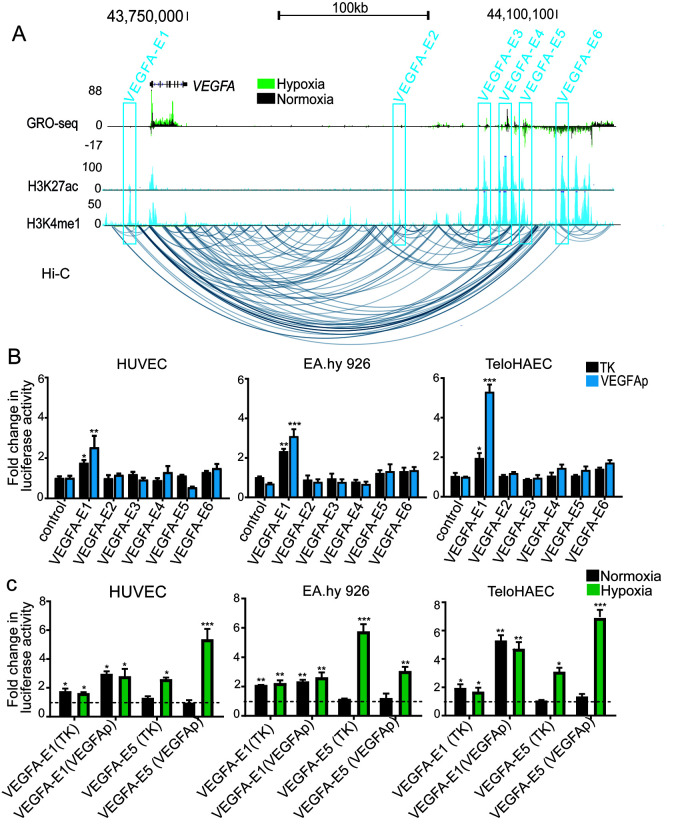
*VEGFA* enhancer identification and reporter assay. (A) UCSC browser image depicting the *VEGFA* locus and six putative enhancers (*VEGFA-E1* to *VEGFA-E6*) in HUVECs. Enhancer regions are highlighted in blue, and arcs below the tracks depict significant interactions from Hi-C data. Coordinates of cloned enhancers are as follows: *VEGFA-E1* (chr6: 43720993 to 43722024), *VEGFA-E2* (chr6: 43940393 to 43941036), *VEGFA-E3* (chr6: 44008921 to 44010132), *VEGFA-E4* (chr6: 44026392 to 44027694), *VEGFA-E5* (chr6: 44040639 to 44041514), and *VEGFA-E6* (chr6: 44072050 to 44073251). (B) Luciferase reporter assay of the activities of the six putative enhancer regions either in the context of the TK minimal promoter or the *VEGFA* promoter. Each reporter construct was transfected into HUVECs, EA.hy926 cells, or TeloHAECs and assayed after 48 h. (C) Activities of *VEGFA* enhancers 1 and 5 (*VEGFA-E1* and *VEGFA-E5*) under hypoxia stimulus. Cells transfected with the *VEGFA-E1* and *VEGFA-E5* vectors and control vectors were incubated under either hypoxia or normoxia, and luciferase activity was assessed after 24 h. All luciferase data are represented as fold changes from firefly luciferase activity (normalized to *Renilla* luciferase activity) induced by enhancer vectors over the firefly signal obtained with their respective control vectors. Error bars represent means ± standard errors of the means (SEM) (*n* = 3). *, *P* value < 0.05; **, *P* value < 0.001; ***, *P* value < 0.0001 (by one-way ANOVA and Dunnett's multiple-comparison test).

To identify putative regulatory elements of the *VEGFA* gene in endothelial cells, we started by identifying genomic loci that displayed enhancer features (defined by H3K27ac and H3K4me1), eRNA expression as determined by GRO-Seq, and chromatin interactions with the *VEGFA* promoter as determined by chromosome conformation capture (Hi-C) data in HUVECs. We identified six putative *VEGFA* enhancers, which were named *VEGFA-E1* to *VEGFA-E6* (Fig. 1A). The closest enhancer to the *VEGFA* transcription start site (TSS) was *VEGFA-E1* (kb −13). The other five putative enhancers, *VEGFA-E2*, *VEGFA-E3*, *VEGFA-E4*, *VEGFA-E5*, and *VEGFA-E6*, were located +250 kb, +270 kb, +290 kb, +305 kb, and +334 kb downstream of the TSS, respectively. To determine the regulatory activities of the putative enhancers, we assessed their ability to induce reporter gene expression either in the context of the endogenous *VEGFA* promoter or a thymidine kinase (TK) minimum promoter. Enhancer activity was evaluated in primary human endothelial cells, HUVECs, and two additional endothelial cell lines, EA.hy926 and immortalized human aortic endothelial cells (TeloHAECs). Of the six characterized enhancers, only *VEGFA-E1* was able to induce significant luciferase expression in all three endothelial cell lines (Fig. 1B). Importantly, *VEGFA-E1* significantly activated the *VEGFA* promoter, as demonstrated by increased luciferase expression, varying between a 2- and a 5.8-fold increase in the different endothelial cells. The remaining putative enhancers (*VEGFA-E2* to *VEGFA-E6*) were unable to induce any notable activation of luciferase expression in endothelial cells.

Assessment of hypoxia-inducible factor 1α (HIF-1α) chromatin immunoprecipitation sequencing (ChIP-Seq) data (41) indicated that *VEGFA-E5* overlaps a HIF-1α binding site, and it may therefore be activated by hypoxia. Cells transfected with either the *VEGFA-E1* or *VEGFA-E5* vector were incubated under hypoxia or normoxia, and luciferase activity was assessed after 24 h. In line with our predictions, *VEGFA-E5* was able to induce significant expression of luciferase following hypoxia, whereas *VEGFA-E1* was not (Fig. 1C). Collectively, two enhancers (*VEGFA-E1* and *VEGFA-E5*) out of the six predicted by a combination of Hi-C and GRO-Seq data were able to induce reporter gene expression, *VEGFA-E1* constitutively and *VEGFA-E5* in response to a hypoxia stimulus. Based on these results, we selected these two enhancers for further analysis.

### Identification and characterization of *VEGFC* enhancers.

**FIG 2:**
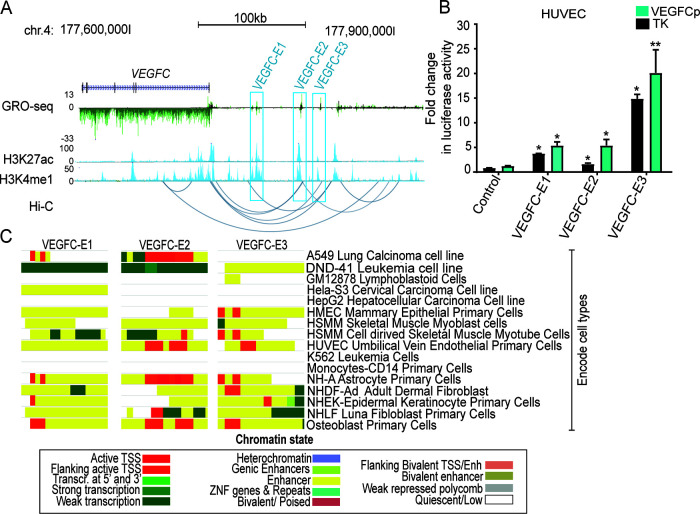
*VEGFC* enhancer identification and reporter assay. (A) UCSC browser image representing the *VEGFC* locus and three putative enhancers (*VEGFC-E1* to *VEGFC-E3*) in HUVECs. Enhancer regions are highlighted in blue, and arcs below the tracks depict significant interactions from Hi-C data. Coordinates of cloned enhancers are as follows: *VEGFC-E1*, chr4 177759862 to 177760852; *VEGFC-E2*, chr4 177804215 to 177805813; and *VEGFC-E3*, chr4 177823631 to 177824876. (B) Luciferase reporter assay of the activities of three identified putative *VEGFC* enhancers in HUVECs. The fold change in luciferase activity was calculated from *Renilla*-normalized firefly luciferase activity induced by enhancer vector constructs in comparisons with the control vectors (TK or *VEGFC* promoters vectors alone). The data are means ± SEM from independent biological repeats (*n* = 3). *, *P* value < 0.05; **, *P* value < 0.001 (one-way ANOVA and Dunnett's multiple-comparison test). (C) Epigenomic chromatin landscape of *VEGFC-E1* to *VEGFC-E3*. Visualization of the chromatin state of enhancer regions across cells was performed in the Washington University Epigenome browser (http://epigenomegateway.wustl.edu/browser/). Transcr., transcription; Enh, enhancer.

Next, we applied the same approach detailed above to identify *VEGFC* enhancers. We identified three candidate enhancers, termed *VEGFC-E1*, *VEGFC-E2*, and *VEGFC-E3*, located 46 kb, 70 kb, and 110 kb upstream of the *VEGFC* TSS, respectively (Fig. 2A). The regulatory activities of these enhancers were also tested in the context of luciferase reporter gene expression in the three different endothelial cell types (data not shown). Results showed that all three enhancers were able to induce luciferase expression in the context of a minimal promoter in HUVECs (Fig. 2B). Among these, *VEGFC-E3* had the highest activation, exhibiting a 6- to a 15-fold increase in reporter gene expression, followed by *VEGFC-E1* (3.7- to 6-fold) and *VEGFC-E2* (1.5- to 2-fold). In addition, both *VEGFC-E3* and *VEGFC-E2* demonstrated further activation in the context of the endogenous *VEGFC* promoter. Previous studies reported histone marks in enhancers to be highly correlated with cell type-specific gene expression (42), and a combination of multiple histone marks allows the characterization of chromatin states (43). Thus, we next compared the chromatin states of the identified *VEGFC* enhancers in different cell types using Encode data. Interestingly, the activities of these enhancers were not limited to endothelial cells but were also present in muscle cells and fibroblasts, among others. However, these enhancers were inactive in other cell types, including monocytes and lymphoblastoid and liver cells (Fig. 2C).

### Validation of *VEGFA* and *VEGFC* enhancer activity at their endogenous loci.

**FIG 3:**
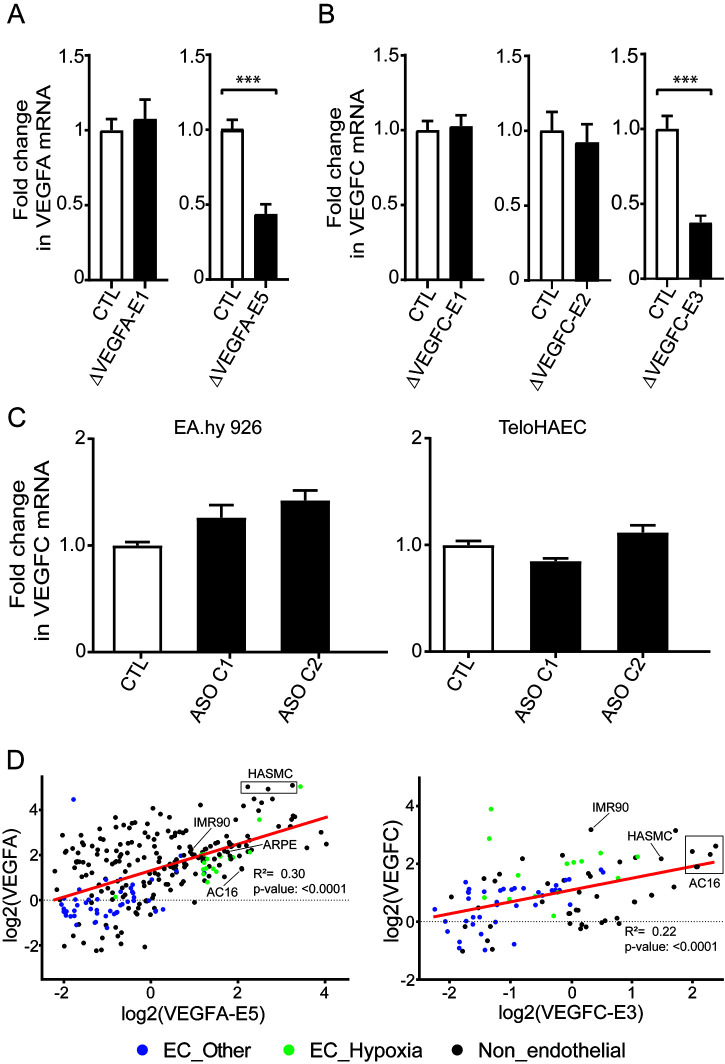
*VEGFA* and *VEGFC* expression upon enhancer deletions. (A) RT-qPCR analysis of *VEGFA* expression in EA.hy926 clones with deletions of *VEGFA-E1* (Δ*VEGFA-E1*) or *VEGFA-E5* (Δ*VEGFA-E5*) and control (CTL) cells that were transfected with only gRNAs. The bar graphs represent the averages of data from 4 and 6 deletion clones of *VEGFA-E1* and *VEGFA-E5*, respectively. The data are represented as means ± standard deviations (SD) (*n* = 3). ***, *P* value < 0.0001 (Student's unpaired two-tailed *t* test). (B) RT-qPCR analysis of *VEGFC* expression in EA.hy926 clones with deletion of *VEGFC-E1* (Δ*VEGFC-E1*), *VEGFC-E2* (Δ*VEGFC-E2*), and *VEGFC-E3* (Δ*VEGFC-E3*). The bar graphs represent the averages of data from 3 and 6 deletion clones of *VEGFC-E1* and *VEGFC-E2*, respectively. For Δ*VEGFC-E3*, no clonal selection was done, as the deletion efficacy was 100%; thus, a pool of transfected cells was considered for expression analysis. To replicate this experiment, two different pairs of gRNAs, gRNA_P1 and gRNA_P2, for *VEGFC-E3* deletion were used. The data are means ± SD (*n* = 3). ***, *P* value < 0.001 (Student's unpaired two-tailed *t* test). (C) RT-qPCR analysis of *VEGFC* expression where eRNA was knocked down using ASOs. ASO C1 and −2 are the different antisense oligonucleotides targeting the *VEGFC-E3* eRNA. The data are means ± SD (*n* = 3). *, *P* value < 0.05; ***, *P* value < 0.0001 (one-way ANOVA and Dunnett's multiple-comparison test). (D) Correlation plot displaying the log_2_ number of reads per kilobase of transcript per million mapped reads (RPKM) of the enhancer *VEGFA-E5* versus that of *VEGFA* and of the enhancer *VEGFC-E3* versus that of *VEGFC* from GRO-Seq data. EC, endothelial cells.

To experimentally validate the functional role of the *VEGFA* (*VEGFA-E1* and *VEGFA-E5*) and *VEGFC* (*VEGFC-E1*, *VEGFC-E2*, and *VEGFC-E3*) enhancers in driving coding gene expression, we used CRISPR/Cas9 to delete the enhancer regions in EA.hy926. By PCR screening and sequencing, we confirmed independent cellular clones carrying homozygous deletions of the target enhancers (data not shown) and characterized their effects on *VEGFA* and *VEGFC* gene expression by quantitative reverse transcription-PCR (RT-qPCR) in different clones. From the results, we did not observe any effect of *VEGFA-E1* deletion on *VEGFA* expression (Fig. 3A), in contrast to the observed *VEGFA-E1* activity in the reporter assays (Fig. 1B). On the other hand, deletion of *VEGFA-E5* resulted in a notable decrease (2.3-fold decrease) in *VEGFA* expression (Fig. 3A). Likewise, *VEGFC-E3* deletion induced a significant decrease in *VEGFC* expression (2.5-fold) (Fig. 3B), in accordance with the reporter assay results (Fig. 2B). Deletion of *VEGFC-E1* and *VEGFC-E2*, on the other hand, did not induce any notable effect on *VEGFC* expression (Fig. 3B). Additional validations using antisense oligonucleotides targeting the *VEGFC* eRNA transcribed from *VEGFC-E3* showed that knockdown of the eRNA did not seem to have an effect on *VEGFC* expression (Fig. 3C), suggesting that the RNA transcript might not be needed for the enhancer effect. Taking these observations together, we conclude that *VEGFA-E5* and *VEGFC-E3* contribute to the endogenous transcriptional expression of *VEGFA* and *VEGFC*, respectively.

To further investigate the role of the *VEGFA-E5* and *VEGFC-E3* enhancers in the regulation of *VEGFA* and *VEGFC* expression, respectively, we correlated the transcriptional activities of the enhancers with their putative target gene across a diverse set of human cells. Previous studies have shown that eRNA expression is highly linked to open chromatin state (indicated by DNase-hypersensitive sites) and regulatory activity (42), allowing evaluation of the chromatin state of the enhancers in cell types that express various levels of *VEGFs* (such as cardiomyocytes, smooth muscle cells, fibroblasts, and epithelial cells). For this, we collected numerous public (44–73) GRO-Seq data sets encompassing 44 cell types across 384 samples and studied the expression of eRNAs from the identified enhancer regions. The results (Fig. 3D) suggest that eRNA expression correlates well with *VEGF* expression throughout cell types and in response to hypoxic stimulation. The results corroborate the functional relevance of these enhancers for *VEGF* expression.

### Nearby lncRNAs play a role in the regulation of *VEGF* gene expression.

**FIG 4:**
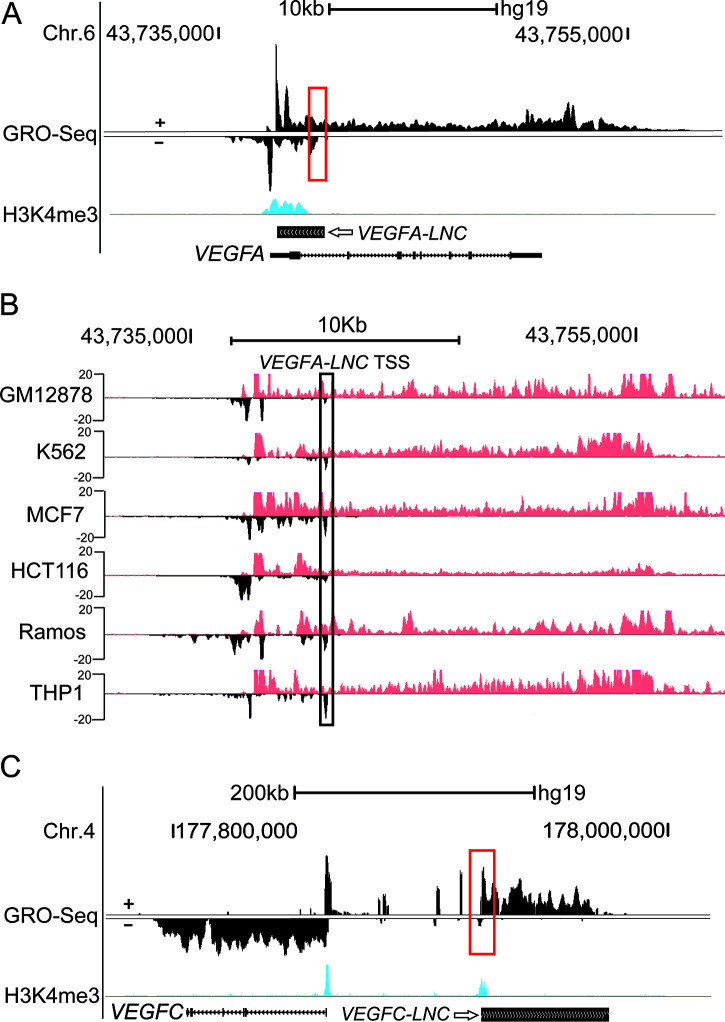
Genomic locations of *VEGFA-LNC* and *VEGFC-LNC*. (A) UCSC browser image depicting normalized GRO-Seq tag densities at the *VEGFA* and *VEGFA-LNC* loci in HUVECs. *VEGFA-LNC* is transcribed in the antisense orientation with respect to the *VEGFA* gene, with its TSS located in an intron ∼4 kb downstream of the *VEGFA* TSS. The deleted promoter region of *VEGFA-LNC* is marked by the upright rectangular box. (B) UCSC browser image representing the *VEGFC* and *VEGFC-LNC* loci in HUVECs. *VEGFC-LNC* is located ∼120 kb upstream of the *VEGFC* TSS. The deleted region of *VEGFC-LNC* is marked by the upright rectangular box. (C) *VEGFA-LNC* and its TSS conserved in different cell types (negative strands; bottom) based on its phyloP score and GRO-Seq signal.

Further assessment of HUVEC GRO-Seq data allowed us to identify other transcripts around the *VEGFA* and *VEGFC* loci. As with the vast majority of mammalian gene TSSs (74), the *VEGFA* and *VEGFC* promoters were characterized by promoter-associated divergent transcription (Fig. 4). In addition, we detected two lncRNAs located nearby the *VEGFA* and *VEGFC* genomic loci, which we named *VEGFA-LNC* and *VEGFC-LNC*, respectively. Similarly to the coding gene TSSs, the lncRNA TSSs were marked by H3K4me3, a histone modification commonly associated with promoter elements. *VEGFA-LNC* is transcribed in the antisense orientation with respect to the *VEGFA* gene, with its TSS located in an intron ∼4 kb downstream of the *VEGFA* TSS (Fig. 4A). Interestingly, *VEGFA-LNC* and its TSS are conserved in many other cell types, such as THP1 monocytes and MCF-7 cancer cells (Fig. 4B). Furthermore, *VEGFA-LNC* was also shown to be expressed in mouse thioglycolate-elicited macrophages (TGM) and C166 mouse endothelial cells, also supporting species conservation (data not shown). We also identified a transcript downstream of *VEGFC-E3* with strong H3K27ac, H3K4me1, and GRO-Seq signals, which we named *VEGFC-LNC*. *VEGFC-LNC* is located ∼120 kb upstream of the *VEGFC* TSS, and it is transcribed in an opposing direction relative to that of the *VEGFC* transcript (Fig. 4C). However, *VEGFC-LNC* was specific to endothelial cells and not conserved (data not shown). Previous studies have shown that lncRNAs can hold coding potential and exert their biological function through small peptides (75). Thus, we next evaluated the coding potential of *VEGFA-LNC* and *VEGFC-LNC* using the CPPred prediction tool (76). CPPred integrates several features, such as open reading frame (ORF) length and coverage and Fickett score, among others, to predict the coding potential. From the results obtained, *VEGFA-LNC* and *VEGFC-LNC* were not predicted to have coding potential (data not shown).

**FIG 5:**
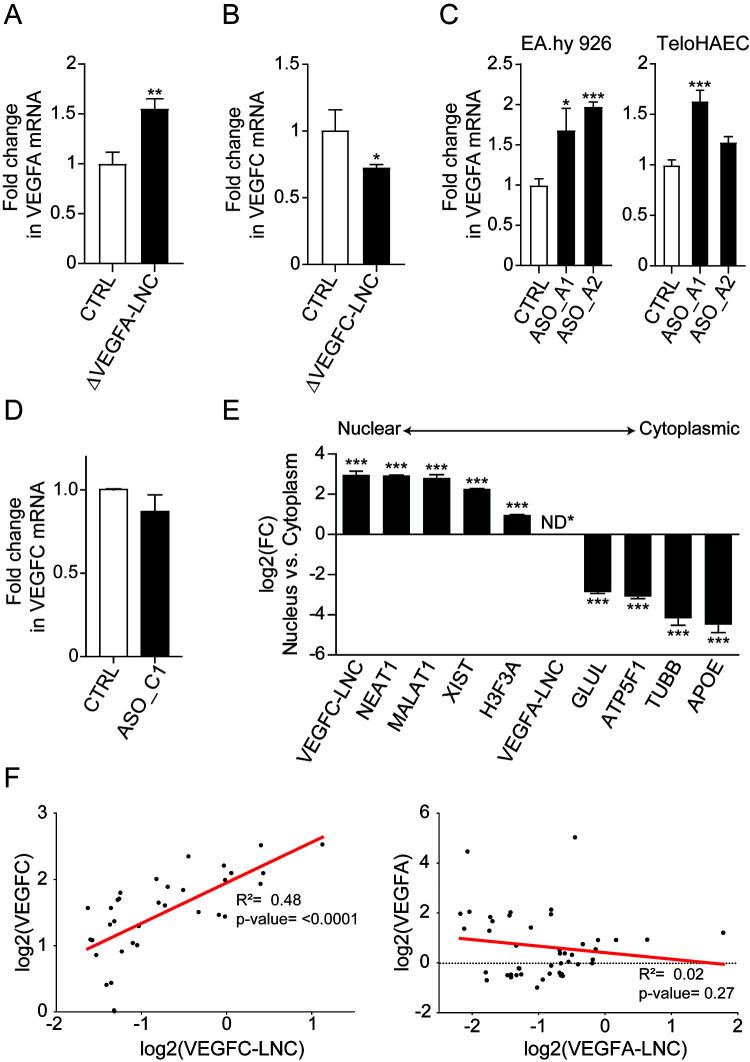
Regulatory function of *VEGFA-LNC* and *VEGFC-LNC.* (A and B) RT-qPCR analysis of *VEGFA* and *VEGFC* expression in control cells or in cell clones where *VEGFA-LNC* (Δ*VEGFA-LNC)* and *VEGFC-LNC* (Δ*VEGFC-LNC*) were deleted. The bar graphs represent the averages of data from 5 deletion clones of *VEGFA-LNC*s or *VEGFC-LNC*. (C) Expression analysis of *VEGFA* in EA.hy926 cells and TeloHAECs where *VEGFA-LNC* was knocked down with an ASO. ASO_A1 and ASO_A2 represent two different ASOs targeting different sites of the *VEGFA-LNC*. Data are represented as means ± SD (*n* = 3). *, *P* value < 0.05; ***, *P* value < 0.0001 (one-way ANOVA and Dunnett's multiple-comparison test). (D) Significance was evaluated with Student’s unpaired, two-tailed *t* test (*n* = 3). (E) Bar plot showing the log_2_ fold change of nuclear versus cytoplasmic fractions of *VEGFC-LNC*, along with other well-known nuclear and cytoplasmic transcripts. Statistical significance was evaluated with Student’s unpaired two-tailed *t* test (*n* = 3). ***, *P* value < 0.001. (F) Correlation plot displaying the log_2_ RPKM of *VEGFA-LNC* versus *VEGFA* or *VEGFC-LNC* versus *VEGFC* from public HUVEC and HAEC GRO-Seq data. ND*, not detected.

To test the regulatory activities of these lncRNAs, we carried out CRISPR/Cas9-mediated deletion of the lncRNA promoters in the EA.hy926 cell line. *VEGFA-LNC* and *VEGFC-LNC* deletions were confirmed by genotyping and RT-qPCR of *VEGFC-LNC* expression levels (data not shown), and confirmed homozygous clones for each lncRNA deletion were chosen for further assessment of the effects on *VEGFA* and *VEGFC* gene expression. RT-qPCR expression analysis in different clones demonstrated that deletion of *VEGFA-LNC* led to a 1.8-fold increase in *VEGFA* mRNA expression (Fig. 5A), whereas a 1.6-fold decrease in the expression of *VEGFC* was observed upon deletion of *VEGFC-LNC* (Fig. 5B). The effects on *VEGF* gene expression were consistent between the clones (data not shown), suggesting that there is minimal clonal variation. Furthermore, degradation of *VEGFA-LNC* with two different antisense oligonucleotides promoted upregulation of *VEGFA*’s expression by 1.7- to 2-fold in EA.hy926 cells and by 1.2- to 1.6-fold in TeloHAECs, supporting the results obtained from *VEGFA-LNC*’s deletion (Fig. 5C), whereas targeting *VEGFC-LNC* with an antisense oligonucleotide did not affect *VEGFC* expression (Fig. 5D), suggesting a transcript-independent effect. Collectively, these data suggest possible roles of *VEGFA-LNC* and *VEGFC-LNC* in transcriptional regulation, participating in the downregulation of *VEGFA* and upregulation of *VEGFC*, respectively.

To determine if the action is predicted to be nuclear and/or cytoplasmic, we next quantified the amount of *VEGFA-LNC* and *VEGFC-LNC* in the nuclear and cytoplasmic fractions in HUVECs using RNA-Seq. The results (Fig. 5E) demonstrated that *VEGFC-LNC* was expressed mainly in the nucleus (average cpm, 8) with other known nuclear lncRNAs (*XIST*, *MALAT1*, *NEAT1*, and *H3F3A*), whereas *VEGFA-LNC* expression was too low to be evaluated (average cpm in the cytoplasm, 0.02). This strongly suggests that *VEGFC-LNC* acts in the nucleus and that *VEGFA-LNC* may play either a role in the cytoplasm or a cotranscriptional role in the nucleus. If the lncRNA regulates transcription in the nucleus, we expect the lncRNA expression in GRO-Seq (which detects nascent RNAs that can also exhibit low stability) to be inversely correlated with *VEGF* expression. To test this, we used all public GRO-Seq data from stimulated HUVECs and HAECs to measure the correlation between the coding gene and lncRNA expression (Fig. 5F). Our results demonstrated a significant positive correlation between *VEGFC-LNC* and *VEGFC* expression, supporting shared mechanisms of transcriptional regulation. On the other hand, a trend of anticorrelation was detected for *VEGFA-LNC* and *VEGFA*, although this was not significant. All together, our results suggest that the activating effect of *VEGFC-LNC* and repressing effect of *VEGFA-LNC* may be due to cotranscriptional *cis* mechanisms.

### Functional evaluation of *VEGFA-LNC* and *VEGFC-LNC* by RNA-Seq.

**FIG 6:**
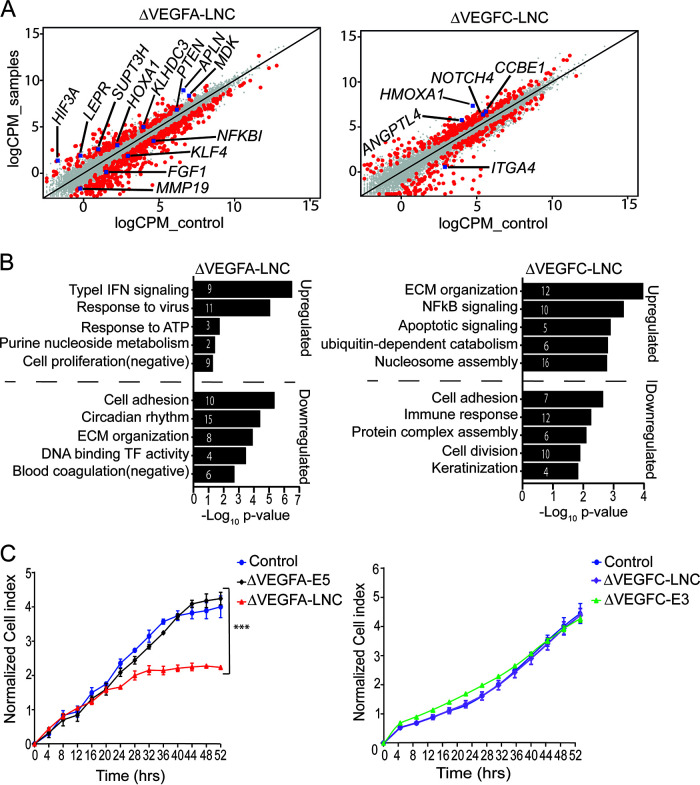
Genome-wide effects of *VEGFA-LNC* and *VEGFC-LNC* deletions. (A) Scatterplot of differentially expressed genes after *VEGFA-LNC* and *VEGFC-LNC* deletion. Genes that exhibited at least a 1.5-fold change and a 0.05 FDR were considered differentially expressed and are indicated by red dots. The blue squares represent differentially expressed genes that have been mentioned in Results. (B) Gene ontology analysis showing biological processes associated with upregulated and downregulated gene sets after *VEGFA-LNC* and *VEGFC-LNC* deletions. Numbers within the bars indicate the number of genes included in the pathways. (C) Proliferation assay of the Δ*VEGFA-LNC*, Δ*VEGFA-E5*, Δ*VEGFC-LNC*, and Δ*VEGFC-E3* constructs (3 clones for each deletion in duplicate wells). The data are means ± SEM (*n* = 3). ***, *P* value <0.0001 (multiple ANOVAs).

To further explore whether the *VEGFA-LNC* and *VEGFC-LNC* deletions had other genome-wide *cis* or *trans* effects, we carried out RNA-Seq on three knockout clonal lines for each region. Deletion of *VEGFA-LNC* resulted in 708 differentially expressed genes (DEGs), of which 484 (68%) were downregulated and 224 (32%) were upregulated (Fig. 6A; see Table S1 in the supplemental material). By the selected thresholds (a fold change [FC] of at least 1.5 but not more than −1.5 and a false-discovery rate [FDR] of less than 0.05), however, *VEGFA* itself was not among the DEGs. We further identified potential *cis* targets among these, such as *NFKBI*, *KLHDC3*, and *SUPT3H*, located within 1 Mb of *VEGFA-LNC*, suggesting that the majority of the downstream effects of *VEGFA-LNC* are likely mediated by *trans* mechanisms.

To test if *VEGFA-LNC* could regulate the expression of these genes (Table S1) by directly interacting within their promoter, we used Triplex Domain Finder software (77) to find regions of DNA binding domains (DBDs) within *VEGFA-LNC* that are able to bind to target promoters via triple-helix formation. One region of *VEGFA-LNC*, spanning bp 2412 to 2464, was predicted to significantly bind within the triplex target DNA site (TTS) of 31 genes via triplexes (*MDGA2*, *SIGLEC15*, *DCLK1*, *CCL26*, *RGS17*, *ADAMTS9*, *DHRS13*, *PRSS35*, *KCNQ3*, *CACNA2D1*, *CACNG6*, *STOX2*, *CCR4*, *CD300C*, *OSCAR*, *VASN*, *TRIM29*, *FAM133A*, *KLHDC8B*, *EFCAB6*, *PRR9*, *LRG1*, *EFR3B*, *KDF1*, *CAPN8*, *GCSAML*, *KSR2*, *IL36A*, *NEDD9*, *HDAC9*, and *GAL3ST4* [data not shown]). Of those genes, 11 were upregulated and 20 downregulated upon *VEGFA-LNC* deletion (Table S1). Interestingly, some of these genes, such as *OSCAR*, *TRIM29*, *RGS17*, *VASN*, *LRG1*, *NEDD9*, *IL36α*, *ADAMTS9*, and *HDAC9*, were previously reported to be involved in regulating proliferation in different cell lines and endothelial cells (78–86).

In order to determine the relevant biological functions affected by the *VEGFA-LNC* deletion, gene ontology (GO) analysis was performed separately to select for upregulated and downregulated genes. In this analysis, redundant GO terms were ignored, and the top-most-informative GO terms were selected based on *P* values. The top five enriched biological terms associated with DEGs upon *VEGFA-LNC* deletion are shown in Fig. 6B. The upregulated genes were involved in functional categories related to the type I interferon (IFN) signaling pathway, virus response, ATP response, purine nucleoside metabolism, and negative regulation of cell proliferation. Among these, we identified *CTBP2*, *MXI1*, *CLMN*, *IFIT3*, *IFITM1*, *PTEN*, *PTK2B*, *SSTR5*, *SPRY1*, and the known factors implicated in VEGF pathways, such as *LEPR* (87), *HIF3A*(GO identifier, 0001944), *PTEN* (88), *LRG1* (82), *MDK* (89) (GO identifier, 0001944), and *HOXA1* (90). On the other hand, downregulated genes were centered on categories related to cell adhesion, circadian rhythm, extracellular matrix (ECM) organization, negative regulation of DNA binding transcription factor activity, negative regulation of blood coagulation, and response to cyclic AMP (cAMP). We were also interested in the identification of potential upstream regulators that may explain the observed differential expression. To do so, we used IPA (Ingenuity Pathway Analysis; Qiagen) to identify both activated and inhibited upstream regulators. The list of top upstream regulators associated with DEGs following *VEGFA-LNC* deletion that we extracted is shown in Table 1.

**TABLE 1:** Top 20 upstream regulators upon deletion of *VEGFA-LNC*

Upstream regulator	Molecule type	Activation state	Z-score	*P* value
IFN-γ	Cytokine	Activated	2.2	2.41E–19
IRF7	Transcription regulator	Activated	4.1	1.84E–14
PRL	Cytokine	Activated	3.8	8.00E–14
IFN-α (group)	Cytokine	Activated	3.1	2.69E–13
TGFB1	Growth factor	Inhibited	–4.6	1.50E–12
STAT2	Transcription regulator	Activated	2.1	2.64E–12
STAT1	Transcription regulator	Activated	2.7	5.75E–12
IL1RN	Cytokine	Inhibited	−2.9	8.92E–12
IFNA2	Cytokine	Activated	3.5	3.26E–10
PML	Transcription regulator	Activated	3.9	2.14E–09
TREM1	Transmembrane receptor	Inhibited	–4.1	3.90E–09
BTK	Kinase	Inhibited	–3.2	5.54E–09
SMAD4	Transcription regulator	Inhibited	–3.1	1.21E–08
IFNA1/IFNA13	Cytokine	Activated	2.7	1.67E–08
IRF1	Transcription regulator	Activated	2.7	1.23E–07
ATXN3	Peptidase	Activated	2.0	5.19E–07
PD98059	Kinase inhibitor	Activated	2.0	5.40E–07
IRF8	Transcription regulator	Inhibited	−2.8	7.17E–07
F2	Peptidase	Inhibited	−2.8	1.19E–06

The top 5 upstream regulators that appeared on this list include three cytokines (IFN-γ, IFN-α, and prolactine [PRL]), one transcription factor (IRF7), and one growth factor (transforming growth factor β1 [TGFB1]). All together, our results suggest that some of the downstream effects of *VEGFA-LNC* deletion may be mediated by *VEGFA*’s altered expression, but the majority may act through other pathways, such as interferon signaling.

The same criteria described above were applied to study the DEGs upon *VEGFC-LNC* deletion (Fig. 6A; Table S1). Deletion of *VEGFC-LNC* resulted in a total of 520 DEGs, of which 298 (57%) and 222 (43%) were upregulated and downregulated, respectively. We also used Triplex Domain Finder software to find potential DNA binding domains within *VEGFC-LNC* that could bind to the promoters of the DEGs (Table S1). Using this approach, we identified 37 DBD regions; however, none of these were statistically significant. We further performed GO analysis to identify biological processes associated with DEGs upon *VEGFC-LNC* deletion. The top biological terms identified with upregulated genes were associated mainly with extracellular matrix (ECM) organization, NF-κB signaling, apoptotic signaling, ubiquitin-dependent catabolism, and cell adhesion, while downregulated genes were related to nucleosome assembly, immune response, protein complex assembly, cell division, and keratinization (Fig. 6B). Here again, several of these pathway-related genes have been linked to *VEGFC-*mediated effects, including *CCBE1* (91), *ITGA4* (92), *NOTCH4* (93), *ANGPTL4* (94), and *HMOX1* (95). We could not predict any potential *VEGFC-LNC* downstream *cis* target, as the closest gene among the DEGs was ∼5 Mb distant from the *VEGFC-LNC* TSS. Furthermore, we identified several upstream regulators that could explain the observed DEGs upon *VEGFC-LNC* deletion, shown in Table 2. Among these, the TP53 transcription factor was the most significant upstream regulator, and it was predicted to be activated. Similarly, several growth factors, including *TGFB2*, *TGFB1*, *VEGF*, and *EGF*, were predicted to be activated. This suggests that *VEGFC-LNC* may mediate substantial effects through VEGF signaling, although alternative mechanisms may also exist.

**TABLE 2:** Top 20 upstream regulators upon deletion of *VEGFC-LNC*

Upstream regulator	Molecule type	Activation	Z-score	*P* value
TP53	Transcription regulator	Activated	3.61	4.25E–09
TGFB2	Growth factor	Activated	2.90	4.14E–08
TGFB1	Growth factor	Activated	2.59	6.22E–07
LY294002	Kinase inhibitor	Inhibited	−2.53	1.04E–06
TNF	Cytokine	Activated	3.21	2.18E–06
F2	Peptidase	Activated	3.37	2.88E–06
VEGF	Growth factor	Activated	2.17	4.86E–06
EDN1	Cytokine	Activated	2.54	4.93E–06
TP63	Transcription regulator	Activated	2.23	5.92E–06
U0126	Kinase inhibitor	Inhibited	–2.51	9.20E–06
ERK	Kinase	Activated	2.35	2.21E–05
EGF	Growth factor	Activated	2.11	5.93E–05
IFN-β	Cytokine	Activated	2.17	6.72E–05
P38 MAPK	Kinase	Activated	3.09	1.17E–04
JNK	Transcriptional regulator	Activated	2.40	1.43E–04
HIF1A	Transcription regulator	Activated	2.93	1.77E–04
NFAT	Transcription regulator	Activated	2.41	3.31E–04
ATF4	Transcription regulator	Activated	2.15	8.19E–04
EGFR	Kinase	Activated	2.07	1.21E−03
AP-1	Transcription regulator	Activated	2.18	1.24E−03

### Effect of lncRNAs and eRNA deletion on cell proliferation and migration.

In order to investigate whether the identified lncRNAs and eRNA have an effect on cell proliferation and migration and to confirm the RNA-Seq data of the lncRNA-deleted clones, we performed cell proliferation and migration assays. Proliferation assay results demonstrated that deletion of *VEGFA-LNC* significantly reduced cell proliferation (Fig. 6C), while *VEGFC-LNC* and enhancer deletions did not show any notable effect on cell proliferation (Fig. 6C). These results support our findings from the RNA-Seq analysis of *VEGFA-LNC* deletion, where a negative effect on cell proliferation was observed among biological processes associated with upregulated genes upon deletion. On the other hand, scratch wound assays did not show any effect on the cell migration of the clones carrying *VEGFA-LNC* and *VEGFC-LNC* deletions (data not shown).

## DISCUSSION

In our study, we performed in-depth characterization of the genomic loci around *VEGFA* and *VEGFC* genes and identified novel enhancers and lncRNAs that play a role in their expression in endothelial cells. While the enhancers clearly upregulated gene expression, lncRNAs demonstrated various functions. CRISPR/Cas9 deletion and genome-wide RNA-Seq demonstrated several downstream targets of lncRNAs, including factors related to endothelial functions, such as angiogenesis and cell proliferation.

In this study, more enhancers were predicted from Hi-C and GRO-Seq data than were found to have activity in the reporter assay. A similar discrepancy has previously been reported (96), and enhancer regions with no activity in reporter assays may still have a biological role in gene regulation, acting as seed regions in order to bring important factors in three dimensions (3D) to activate others (97). Furthermore, the identified enhancers induced varied expression of the reporter genes among different cell lines, which can be explained by the different availabilities of transcription factors between cell types (32). Interestingly, Hi-C data also showed interactions among the enhancers, in accordance with previous studies where enhancer interactions were detected within the range of 1 kb to 10 Mb (98). Enhancer interactions have been reported to be cooperative and exhibit eRNA expression correlation as well as target gene correlation with the number of enhancers. Therefore, it was interesting to note that not all enhancers that were functionally active in the context of the reporter assay displayed endogenous activity on their target genes. To this end, only deletion of *VEGFA*-associated enhancer (*VEGFA-E5*) and *VEGFC*-associated enhancer (*VEGFC-E3*) resulted in the downregulation of their target genes. This may be due to the episomal context of the reporter plasmids, which does not recapitulate the long-range regulatory interactions of the endogenous chromatin (99). Supporting this, *VEGFA-E5*’s endogenous effect on *VEGFA* did not require hypoxia, contrasting with the reporter assay results. In addition, the resolution of 10 kB of the Hi-C data constitutes a potential limitation of our study, making some interactions, such as those between the enhancer *VEGFC-E1* and the *VEGFC* promoter, not evident. Thus, future studies are needed to better understand the cooperativity and hierarchy of enhancers activating *VEGF* expression.

Interestingly, enhancers *VEGFA-E5* and *VEGFC-E3* were not the closest ones to their respective gene promoters, in accordance with the enhancer hallmark that they can act independently of their distance from and orientation to the target genes and exert their function at large distances by looping mechanisms (100). Additionally, knockdown of eRNA transcribed from *VEGFC-E3* with antisense oligonucleotides (ASOs) did not result in any notable effects in *VEGFC* expression, suggesting that the transcript itself is not required for transcriptional regulation. This is in line with previous findings where instead of the RNA transcript itself, the DNA regulatory elements were found to be more important for function (96, 101).

Our study has also identified two lncRNAs, *VEGFA-LNC* and *VEGFC-LNC*, that may provide additional mechanisms regulating *VEGFA* and *VEGFC* expression, respectively, in endothelial cells. We showed that deletion of *VEGFA-LNC* resulted in a transcriptional increase in *VEGFA*, suggesting that *VEGFA-LNC* may participate in the transcriptional interference of the *VEGFA* locus. In line with previously reported data, lncRNAs can induce transcription interference by displacement of transcription factors near the promoter, nucleosome reposition over the promoter, and obstruction of RNA polymerase II (Pol II) (17, 23, 102). Interestingly, *VEGFA-LNC* was also conserved in mouse cells, suggesting its potential evolutionary importance in *VEGFA* regulation. Upregulation of *VEGFA* expression would be beneficial for the treatment of diseases that would benefit from angiogenesis, and this could be achieved by targeting *VEGFA-LNC*. *In vivo* studies using ASOs directed to lncRNAs have already achieved successful results in reducing multiple myeloma (103); thus, ASO targeting of *VEGFA-LNC* opens up new therapeutic possibilities.

A recent study also identified two short antisense *VEGFA* lncRNAs that regulate *VEGFA* expression in hypoxia, which were shown to be upregulated along with *VEGFA* and localized to its promoter and upstream elements (104). Currently, it is unclear whether these lncRNAs are spliced from the longer *VEGFA-LNC* described here, which initiates transcription from the 1st intron of the *VEGFA* gene, or whether these lncRNAs represent different transcripts. However, supporting the latter option, knockdown of these *VEGFA* lncRNAs with ASOs downregulated *VEGFA* expression, suggesting a mechanism of action that is opposite from that of *VEGFA-LNC* (104).

Analysis of RNA-Seq data from *VEGFA-LNC* or *VEGFC-LNC* deletions identified differentially expressed genes on the same and different chromosomes, indicating large genome-wide effects. Importantly, none of the observed differentially expressed genes coincided with the off-target genes predicted for the guide RNAs (gRNAs) used. The genome-wide effects induced by lncRNAs previously reported include modulation of target gene expression in close proximity to *cis*-acting regulatory mechanisms or elsewhere in the genome via *trans*-acting mechanisms (23, 25). In order to differentiate among these options, we used ASOs to perform a knockdown of *VEGFA-LNC* and found that *VEGFA* was upregulated almost at the same activation level as achieved in the *VEGFA-LNC* deletion clonal cell lines. Indeed, this finding suggests that the regulatory activity of *VEGFA-LNC* on *VEGFA* may be mediated by a *cis* mechanism that is dependent on the RNA transcript itself. However, a recent study reported that ASOs targeting the proximal region of a transcript can also trigger premature termination (105), thus providing an alternative explanation for the results. In support of the cotranscriptional role of *VEGFA-LNC*, we found *VEGFA-LNC* transcript levels to be nearly undetectable in regular RNA-Seq analyses, which, unlike with GRO-Seq, reflects the pool of stable RNAs in a cell. This suggests that the large *trans* effects evidenced by the majority of the downstream target genes being located on different chromosomes may be driven by the *cis* effects on *VEGFA*, *NFKBIE*, *KLHDC3*, and *SUPT3H*. For example, *NFKBIE* and *VEGFA* have been previously shown to be associated with the type I IFN signaling pathway (106, 107), which was the top gene ontology category induced by *VEGFA-LNC* promoter deletion. Moreover, type I IFNs have been shown to inhibit endothelial cell proliferation, another of the top-regulated categories after *VEGFA-LNC* deletion (108). Still, we cannot rule out the possibility that for the 5% of the genes regulated by *VEGFA-LNC*, the effect is mediated by the transcript itself. In fact, lncRNAs can exert their function by regulating chromatin topology, by acting as a scaffold, or by acting as decoys of proteins, such as chromatin-remodeling complexes or transcription factors and other RNAs, such as microRNAs (miRNAs) (109). Interestingly, among the differentially expressed genes predicted to directly interact with *VEGFA-LNC*, we found genes known to participate in cell proliferation and endothelial function. Among these, *ADAMTS9* has been shown to act as an endogenous angiogenesis inhibitor (110, 111). In addition, the role of *HDAC9* has been studied in HUVECs, with which overexpression and knockdown studies reduced and increased the tube formation and sprouting capacity of the cells, respectively (86, 112). Moreover, a positive-feedback loop regulation among the *HDAC9* and *VEGFA* genes has been proposed in previous studies, and *in vivo* animal models confirmed the role of *HDAC9* in vessel formation (86). Regulation of *ADAMTS9* and *HDAC9* expression may thus represent mechanisms by which *VEGFA-LNC* affects cellular proliferation, which warrants future studies.

Unlike with *VEGFA-LNC*, deletion of *VEGFC-LNC* decreased the transcription of *VEGFC* expression, suggesting a transcriptional activation of *VEGFC* by *VEGFC-LNC*. This may be due to *VEGFC-LNC* interacting with chromatin remodeling factors and transcription factors, thereby inducing expression of nearby or distant genes (23, 113). In line with this, our RNA-Seq data showed that *VEGFC-LNC* is predominantly expressed in the nucleus, supporting the hypothesis that it may directly act on the *VEGFC* locus. Alternatively, the DNA regulatory elements may be important for *VEGFC* expression (96). Indeed, *VEGFC-LNC* knockdown did not affect *VEGFC* expression, suggesting that the transcript itself is not required to mediate the effect. Certainly, the *VEGFC* gene, enhancers, and the lncRNA are all located within the same regulatory space based on Hi-C data, and the removal of any of the components of the regulatory hub can lead to a decrease in transcriptional output (114). Inhibition of *VEGFC* expression may be beneficial for the treatment of diseases with excess vascularization, such as cancer. Furthermore, we have also noticed the presence of an lncRNA next to the *KDR* gene (data not shown), suggesting that this may be a widely used mechanism within VEGF family members.

To date, a number of lncRNAs linked to endothelial biology have been characterized (115); they regulate cell cycle control, induction of apoptosis, chromatin remodeling, induction of angiogenesis, and cell adhesion. In line with this, factors linked to cell adhesion were found among both *VEGFA-LNC* and *VEGFC-LNC* downstream target genes, and apoptosis signaling linked factors were distinctively associated with *VEGFC-LNC* downstream targets. In addition, among both *VEGFA-LNC* and *VEGFC-LNC* downstream pathway genes, some were linked to *VEGFA* and *VEGFC* pathways, such as those involved in angiogenesis and lymphangiogenesis. On the other hand, negative regulation of cell proliferation was found to be affected by a subset of genes upregulated upon *VEGFA-LNC* deletion. Together, these data suggest a possible implication of *VEGFA-LNC* and *VEGFC-LNC* in endothelial biology.

In conclusion, this study uncovers novel insights into the regulatory landscape of lncRNAs of *VEGFA* and *VEGFC* in endothelial cells. In this study, by integrating Hi-C and GRO-Seq data as well as reporter assays and genomic deletions, we identified novel as well as functional enhancers and lncRNAs that regulate *VEGFA* and *VEGFC.* These previously unknown *VEGFA* and *VEGFC* regulatory elements might be targeted to modulate *VEGFA* and *VEGFC* expression in endothelial cells. We provide a better understanding of the complex interplay of ncRNAs with gene regulation that might open up novel therapeutic approaches in the future.

## MATERIALS AND METHODS

### Enhancer identification and vector construction.

Chromosome conformation capture (Hi-C) and global run-on sequencing (GRO-Seq) data from HUVECs (44) were used to locate genomic regions that interact with the *VEGFA* and/or *VEGFC* promoters and to demonstrate eRNA transcription, respectively. Putative candidate enhancers, together with *VEGFA* and *VEGFC* promoters, were amplified from HUVEC genomic DNA by PCR with Phusion Hot Start II DNA polymerase (Thermo Fisher Scientific, Waltham, MA) and specific primers. *VEGFA* enhancers 1 and 5 (*VEGFA-E1* and *VEGFA-E5*) were amplified using the following forward (FW) and reverse (RV) primer pairs (sequences are 5′ to 3′): *VEGFA-E1*-FW, GGGTGGGCCTAGTTAGTGCT; *VEGFA-E1*-RV, CCTGTGCTAGGGGATGGAAAT; *VEGFA-E5*-FW, CAGAGGGGTCAAACAACTGG; and *VEGFA-E5*-RV, GCAGGGAGCTGGTCTGTTT. *VEGFC* enhancers 1 (*VEGFC-E1*), 2 (*VEGFC-E2*), and 3 (*VEGFC-E3*) were amplified using the primer pairs *VEGFC-E1*-FW (ACCGCAATTGTGATGTTTGGA), *VEGFC-E1*-RV (AACCCTTCTGAACCTCACGT), *VEGFC-E2*-FW (AAGGCTGGGCAGATTCTACA), *VEGFC-E2*-RV (TGACTACGAAATGGGAATTGGA), *VEGFC-E3*-FW (AAGGAACAGTTTACATAGGTCACG), and *VEGFC-E3*-RV (CAGACAGGTCCTTGCTGTATATTT). *VEGFA* and *VEGFC* promoters (chromosome 6 [chr6] 43735395 to 43738046 and chr4 177713777 to 177717239, respectively) were cloned into the Kpn/EcoRV sites of the pGL4.10 (Promega, Madison, WI) firefly luciferase vector to produce pGL4.10-VAp and pGL4.10-VCp, respectively. The candidate enhancers were subsequently cloned into the SalI site of pGL4.10-VAp or the pGL4.10-VCp vector and into pGL4.10-TK, which contained the thymidine kinase (TK) minimum promoter that drives the expression of the firefly luciferase reporter gene.

### Cell lines and maintenance.

The human endothelial cell lines EA.hy926 (CRL-2922; ATCC, Manassas, VA) and TeloHAEC (CRL-4052; ATCC) were maintained in Dulbecco’s modified Eagle’s medium (DMEM)-high glucose (D5671; Sigma, Saint Louis, MO) supplemented with 10% fetal bovine serum (FBS) and 1% penicillin-streptomycin and in vascular cell basal medium (PCS-100-030; ATCC) supplemented with a vascular endothelial cell growth kit (PCS-100-041; ATCC), respectively. HUVECs were isolated by collagenase digestion (116) from umbilical cords obtained from the maternity ward of the Kuopio University Hospital, with approval from the Research Ethics Committee of the Northern Savo Hospital District. Prior written consent was obtained from the participants, and the experiments were performed according to the relevant regulations and the Declaration of Helsinki (117). HUVECs were cultured on fibronectin (10 g/ml)-gelatin 0.05% (Sigma)-coated culture plates and maintained in endothelial cell growth medium (EGM) supplemented with EGM SingleQuots (CC-3124; Lonza, Basel, Switzerland). Hypoxia was induced by incubating the cells in a Ruskinn InVivo2 400 hypoxia incubator in the presence of 1% O_2_ and 5% CO_2_.

### Transfection and reporter assay.

To perform transfection, cells were seeded into 96-well cell culture plates (PerkinElmer, Turku, Finland) at a density of 1.2 × 10^4^ cells per well and transfected 1 day later with Lipofectamine 3000 (Thermo Fisher Scientific) in antibiotic-free medium. Individual wells were cotransfected with 90 ng of an individual enhancer vector construct and 10 ng of a *Renilla* luciferase expression vector (pGL4.75 [hRluc/CMV]; Promega), which was used for normalization. Transfected cells were maintained in their specific growth media, and a luciferase reporter assay was performed 48 h after transfection using a dual-luciferase reporter assay system (E1910; Promega). The luciferase activity induced by a candidate enhancer was measured using a CLARIOstar microplate reader (BMG Labtech, Ortenberg, Germany). The luciferase signal was first normalized to the *Renilla* luciferase signal and subsequently to the signal obtained from the control plasmid (plasmid without the enhancer).

### CRISPR/Cas9-mediated enhancers and lncRNA deletion.

CRISPR/Cas9-mediated deletion of target regions was performed using the Alt-R CRISPR-Cas9 system (Integrated DNA Technology [IDT], Coralville, IA). Briefly, CRISPR/Cas9 single gRNAs (Table 3) flanking the target enhancers and lncRNAs were designed using an online CRISPR design tool (http://chopchop.cbu.uib.no) and ordered from IDT as CRISPR RNAs (crRNAs). These crRNAs were annealed to a fluorescently labeled *trans*-activating CRISPR RNA (tracrRNA; ATTO 550 no. 1075927; IDT) and complexed with Cas9 endonucleases (HiFi Cas9 nuclease V3, no. 1081060; IDT) to form the ribonucleoprotein complex (RNP). The RNP complexes were then delivered into EA.hy926 cells by reverse transfection using Lipofectamine CRISPRMAX reagent (CMAX00003; Thermo Fisher Scientific) by following the IDT genome-editing protocol. Two days later, transfected cells were harvested and assayed with flow cytometry (CytoFLEX) to determine the transfection efficiency and thereafter diluted for clone selection. Cell medium was refreshed every 4 days until single discrete colonies were formed, picked, and expanded for genotyping and expression analysis. As the negative control, the cells were transfected with gRNAs without Cas9 proteins, and they went through the same clonal selections as actual deletions for 2 weeks. In order to analyze enhancers and lncRNA deletions of EA.hy926 clones, genomic DNA was extracted using a NucleoSpin tissue kit (Macherey-Nagel, Düren, Germany) and amplified by PCR using specific primers (Table 3) flanking the deletion sites and PCR master mix (K0172, 2×; Thermo Fisher Scientific). PCR products were then analyzed by electrophoresis in 1% agarose gel. The deletion was further confirmed by Sanger sequencing of the PCR product.

**TABLE 3:** List of gRNA sequences and genotyping primers used in CRISPR deletions

Primer name	Sequence (5′→3′)
VEGFA-E1_left (gRNA)	GACATGATCAACCCTATAGA
VEGFA-E1_right (gRNA)	GGTGAGCAGGGGCTATACAT
VEGFA-E5_left (gRNA)	GAGAGCCAGGATGCACAGTG
VEGFA-E5_right (gRNA)	GTCGAGTGGCGCAGAGGAGC
VEGFC-E1_left (gRNA)	GTATTGTGACTTGCACAGAC
VEGFC-E1_right (gRNA)	AGGTGTCTGTCATAAATCCT
VEGFC-E2_left (gRNA)	GAGTGCAAATAAGTCCTATT
VEGFC-E2_right (gRNA)	TGTAAGCCTTAACCATACCA
VEGFC-E3_left_p1 (gRNA)	TCTCTAACACTATTAGAACT
VEGFC-E3_right_p1 (gRNA)	TGATGCACTACAATTCTCTC
VEGFC-E3_left_p2 (gRNA)	TGTGCAGTGGACTGAACCAC
VEGFC-E3_right_p2 (gRNA)	TGCATCAAGCCTGGATAAAT
VEGFA-LNC_left (gRNA)	GAATAACCCAGCATGCCCAC
VEGFA-LNC_right (gRNA)	CGGAACACCAGACCCTGCTA
VEGFC-LNC_left (gRNA)	GATGTATCGTAATGCTAAGC
VEGFC-LNC_right (gRNA)	GCTGTGCTAAGGTAATTCAT
VEGFA-E1_del-FW (genotyping)	GGGTGGGCCTAGTTAGTGCT
VEGFA-E1_del-RV (genotyping)	CCTGTGCTAGGGGATGGAAAT
VEGFA-E5_del-FW (genotyping)	CAGAGGGGTCAAACAACTGG
VEGFA-E5_del-RV (genotyping)	GCAGGGAGCTGGTCTGTTT
VEGFC-E1_del_FW (genotyping)	ACCGCAATTGTGATGTTTGGA
VEGFC-E1_del_RV (genotyping)	AACCCTTCTGAACCTCACGT
VEGFC-E2_del_FW (genotyping)	AAGGCTGGGCAGATTCTACA
VEGFC-E2_del_RV (genotyping)	TGACTACGAAATGGGAATTGGA
VEGFC-E3_del_FW (genotyping)	AAGGAACAGTTTACATAGGTCACG
VEGFC-E3_del_RV (genotyping)	CAGACAGGTCCTTGCTGTATATTT
VEGFA-LNC_FW (genotyping)	GTACCTGAGTGGGGTGCATT
VEGFA-LNC_RV (genotyping)	GGACTAGGGGCGAGAAAAAC
VEGFC-LNC_FW (genotyping)	TTGTGGGAAGGGAGGAGAAG
VEGFC-LNC_RV (genotyping)	ACAGGCTTAAATGGGAAAATCAG

### ASOs.

Antisense oligonucleotides (ASOs) are short single-stranded DNA oligonucleotides that target RNA by complementarity, leading to RNase H-mediated degradation of the target RNA. ASOs were designed to target 2 different regions of the *VEGFA* lncRNA (ASO A1 sequence [5′→3′, TCTGTCGTCTTAGGTG] and ASO A2 sequence [5′→3′, GAAAGATGGACAGTGG]), belonging to intronic regions of the *VEGFA* gene; two regions of the *VEGFC* enhancer 3 eRNA, one for the forward strand (ASO *VEGFC-E3*-1 sequence [5′→3′, AACTTAGGAATCATAA]) and one for the reverse strand of the eRNA (ASOs *VEGFC-E3*-2 sequence [5′→3′, GATGGTTAAACAAAGC]), and one region of the *VEGFC* lncRNA (ASO C1 sequence [5′→3′, GAGCACGCAGGAAGCT]). ASO control B (LG00000002-FDA; Qiagen, Hilden, Germany) was used as a negative control. EA.hy926 cells and TeloHAECs were seeded into 12-well plates and transfected at 70% confluence with GapmeR LNA ASOs (Qiagen). ASOs were diluted in serum-free medium and transfected into EA.hy926 cells and TeloHAECs at a final concentration of 100 nM, using 3 μl of Lipofectamine 3000 (Thermo Fisher). Cells were washed, and 400 μl of serum-free medium was added with 100 μl of the transfection complex. The medium was changed after 4 h, and cell lysis was performed 48 h after the transfections. RNA and reverse transcription (RT) followed by quantitative real-time PCR (qPCR) were performed as described in the “RNA isolation and expression analysis” section below, but DNase treatment was done with DNase I (Thermo Scientific) instead.

### TDF analysis.

The triplex-forming potential between the lncRNAs and the top 80 differentially expressed genes upon CRISPR deletions was evaluated using Triplex Domain Finder (TDF) (77). A promoter test was used to identify DNA-binding domains (DBDs) within the lncRNAs with the potential to form triple helices in the promoter regions of candidate target genes (referred to as TTS, a triplex target DNA site). The promoter test compares the binding events of the DBD within the promoters of candidate genes (target promoters) with the binding events in the remaining promoters of the genome (nontarget promoters). Default settings, with the hg19 genome and triple helix binding size (–l) set to 20, were used.

### RNA isolation and expression analysis.

Total RNA was isolated from the cells using an RNeasy minikit (74104; Qiagen) and DNase treated with the RNase-free DNase set (79254; Qiagen). RNA was reverse transcribed with random primers using a RevertAid first-strand cDNA synthesis kit (K1621; Thermo Fisher Scientific). Expression analysis was achieved (by RT-qPCR) using a human *VEGFA* (Hs00900055_m1; Thermo Fisher Scientific) and human *VEGFC* (Hs01099203_m1; Thermo Fisher Scientific) TaqMan assay or specific primers for *VEGFC-LNC* (5′→3′, FW, CCAGGAGCCTCAAACTCTAATC; and RV, TGCTGCATCCTTGTCCTTAA) with the FastStart Universal SYBR green master (Rox) assay (4913914001; Merck). Gene expression was normalized to that of *GAPDH* or *ACTB* (4333764F and 4333762T, respectively; Applied Biosystems, Foster City, CA), and relative expression was calculated using the ΔΔ*C_T_* method (where *C_T_* is threshold cycle) (118).

### RNA library preparation and sequencing.

Prior to RNA library preparation, the quality of the input RNA was assessed by Fragment Analyzer (Agilent Technologies, Santa Clara, California) using an RNA kit (DNF-471; Agilent Technologies) and a RNA quality number [RQN] threshold of >9. RNA library preparation was carried out using a NEBNext Ultra II directional RNA library prep kit for Illumina (E7765; New England BioLabs, Ipswich, MA) according to the manufacturer’s instructions. Briefly, 0.5 μg of DNase-treated RNA was subjected to rRNA depletion, followed by RNA fragmentation, cDNA synthesis, and 11 cycles of PCR amplification. The resultant library was quantified using a Qubit double-stranded-DNA HS assay kit (Q32851; Thermo Fisher Scientific), and its quality was checked with a Bioanalyzer (Agilent Technologies). Individual libraries were pooled in equimolar amounts (4 nM for each) and sequenced with the NextSeq 550 sequencer (Illumina, San Diego, CA) using 75 single-end cycles for 10 million reads per sample.

### RNA-Seq and GRO-Seq data analysis.

For RNA-Seq gene expression quantification of *VEGFA-LNC* and *VEGFC-LNC* knockouts, raw reads were trimmed and filtered using Trim Galore (v.0.4.4) with a Phred quality score cutoff of 30. Processed reads were aligned to the GRCh37 genome assembly using STAR version 2.5.4b (119) with the options outFilterMismatchNoverLmax 0.04 and outFilterMultimapNmax 10. Aligned reads mapping to features were assigned using featureCounts (Rsubread 1.32.4) with the Gencode v19 gene transfer format (GTF) file. Transcripts with low cpm of <1 not present in at least 2 libraries above this threshold were considered not expressed and removed from the analysis. Library sizes were subsequently normalized using TMM, and quasi-likelihood F-testing was used to estimate differential expression using edgeR (3.24.3). Transcripts with a FC of at least 1.5 but not more than −1.5 and a false-discovery rate (FDR) of <0.05 were considered differentially expressed. Gene ontology analysis of differential expressed genes (DEGs) was achieved using the Database for Annotation Visualization and Integrated Discovery (DAVID), v6.8 (120). The IPA (Ingenuity Pathway Analysis; Qiagen) tool was applied for identification of canonical pathways associated with DEGs and for analysis of upstream regulators. The prediction of upstream regulators was based on an overlap of both the *P* value and Z-score. Hence, thresholds of an absolute Z-score of |≥|2 and a *P* of <0.05 were set for significant upstream regulator selection.

For RNA-Seq gene expression of HUVEC nuclear and cytoplasmic fractions, the nf-core RNA-Seq pipeline with standard options was used (121). Tag directories were created for each replicate using HOMER v4.10 (122) with the makeTagDirectory.pl command and “-flip” option. Raw counts were quantified using analyzeRepeats.pl with “-strand +,” “-noadj,” and “-count genes” (to quantify noncoding transcripts in nuclear and cytoplasmic fractions, *XIST*, *MALAT1*, *NEAT1*, *VEGFC-LNC*, and *VEGFA-LNC*) or “-count exons” (to quantify coding transcripts in nuclear and cytoplasmic fractions, *H3F3A*, *APOE*, *TUBB*, *ATP5F1*, and *GLUL*), using a custom annotation file containing the coordinates of our lncRNAs of interest.

For GRO-Seq analysis, public data sets (shown in Table 4) were quantified with HOMER v4.10 (122) using analyzeRepeats.pl with the “-strand +,” “-rpkm,” and “-count genes” options and a custom annotation file containing the coordinates of our enhancers (*VEGFA-E5* and *VEGFC-E3*) and lncRNAs (*VEGFA-LNC* and *VEGFC-LNC*) of interest. For eRNAs, both strands were quantified, and the sum of the counts was used for further analysis. Samples where transcripts were expressed (above 0.2 RPKM in both the eRNA/lncRNA and *VEGF* transcripts) were reported.

**TABLE 4:** Public data sets used in this study

Accession no.	Data set	Original publication
GSE39089	ChIP-Seq (HIF1a)	Mimura et al. (41)
GSE29611	ChIP-Seq (H3K27ac and H3K4me1)	Dunham et al. (18)
GSE52642	Hi-C and GRO-Seq	Kaikkonen et al. (45)
GSE92375	GRO-Seq	Bouvy-Liivrand et al. (46)
GSE48759	GRO-Seq	Kaikkonen et al. (123)
GSE136813	GRO-Seq	Linna-Kuosmanen et al. (47)
GSE118530	GRO-Seq	Moreau et al. (48)
GSE94872	GRO-Seq	Niskanen et al. (44)
GSE103530	GRO-Seq	Kuosmanen et al. (124)
GSE13518	GRO-Seq	Core et al. (49)
GSE27823	GRO-Seq	Wang et al. (50)
GSE38140	GRO-Seq	Galbraith et al. (51)
GSE39878	GRO-Seq	Wang et al. (52)
GSE41009	GRO-Seq	Sigova et al. (53)
GSE41323	GRO-Seq	Danko et al. (54)
GSE41324	GRO-Seq	Danko et al. (54)
GSE43070	GRO-Seq	Jin et al. (55)
GSE51225	GRO-Seq	Luo et al. (56)
GSE51633	GRO-Seq	Liu et al. (57)
GSE53964	GRO-Seq	Allen et al. (58)
GSE60454	GRO-Seq	Core et al. (59)
GSE62046	GRO-Seq	Andersson et al. (60)
GSE62296	GRO-Seq	Meng et al. (61)
GSE66448	GRO-Seq	Niskanen et al. (62)
GSE83860	GRO-Seq	Malinen et al. (63)
GSE84432	GRO-Seq	Toropainen et al. (64)
GSE67519	GRO-Seq	Teppo et al. (65)
GSE67540	GRO-Seq	Heinäniemi et al. (66)
GSE101803	GRO-Seq	Kourtis et al. (67)
GSE96859	GRO-Seq	Franco et al. (68)
GSE102819	GRO-Seq	Gao et al. (69)
GSE86165	GRO-Seq	Andrysik et al. (70)
GSE91011	GRO-Seq	Williamson et al. (71)
GSE67295	GRO-Seq	Stender et al. (72)
GSE117086	GRO-Seq	Viiri et al. (73)

### Proliferation assay.

Cell proliferation was assessed with the real-time cell analyzer multiplate (RTCA MP) instrument (xCELLigence; ACEA Biosciences, Inc.). Briefly, three clones of EA.hy926 Δ*VEGFA-LNC*, Δ*VEGFC-LNC*, Δ*VEGFA-E5*, and Δ*VEGFC-E3* were seeded in E-plate 16 plates (ACEA Biosciences, Inc.) in duplicate wells at a density of 5 × 10^3^ cells/well in a total volume of 200 μl of culture medium. The plate was subsequently placed in an RTCA MP located in the cell culture incubator, and the change in cell proliferation was recorded every 2 h for a period of 52 h.

### Migration assay.

Several clones of EA.hy926 *ΔVEGFA-LNC* and *ΔVEGFC-LNC* knockouts and control cells were seeded in duplicates at a density of 4 × 10^4^ cells/well in IncuCyte Imagelock 96-well plates (Essen BioScience) 24 h prior to the assay. Wounds were effected with the WoundMaker (Essen BioScience) according to the manufacturer’s instructions. Images of the wounds were acquired every 2 h by Incucyte (Essen BioScience) and analyzed with the IncuCyte ZOOM 96-well scratch wound cell migration software. Relative wound density was automatically calculated by measuring the cell density of the wound region relative to the density outside the wound area.

### Statistical analysis.

Statistical analysis was performed using GraphPad Prism v 5.03. For each experiment three biological replicates were used, and the difference in means was analyzed with one-way analysis of variance (ANOVA) and Dunnett's multiple-comparison test or Student´s unpaired two-tailed *t* test. The statistical significance was defined as a *P* of <0.05 (indicated with an asterisk).

### Data availability.

The RNA-Seq data generated in this study have been submitted to the NCBI Gene Expression Omnibus under accession number GSE141669. The public data sets used in this study are shown in Table 4.
